# West African Man with a Cavitary Pneumonia and Cutaneous Nodule

**DOI:** 10.4269/ajtmh.18-0113

**Published:** 2019-01

**Authors:** Norman L. Beatty, Thomas P. Hanzlik, Elizabeth Connick

**Affiliations:** 1 Division of Infectious Diseases, Department of Medicine, University of Arizona College of Medicine, Tucson, Arizona;; 2 Department of Medicine, University of Arizona College of Medicine, Tucson, Arizona

**Figure 1.**
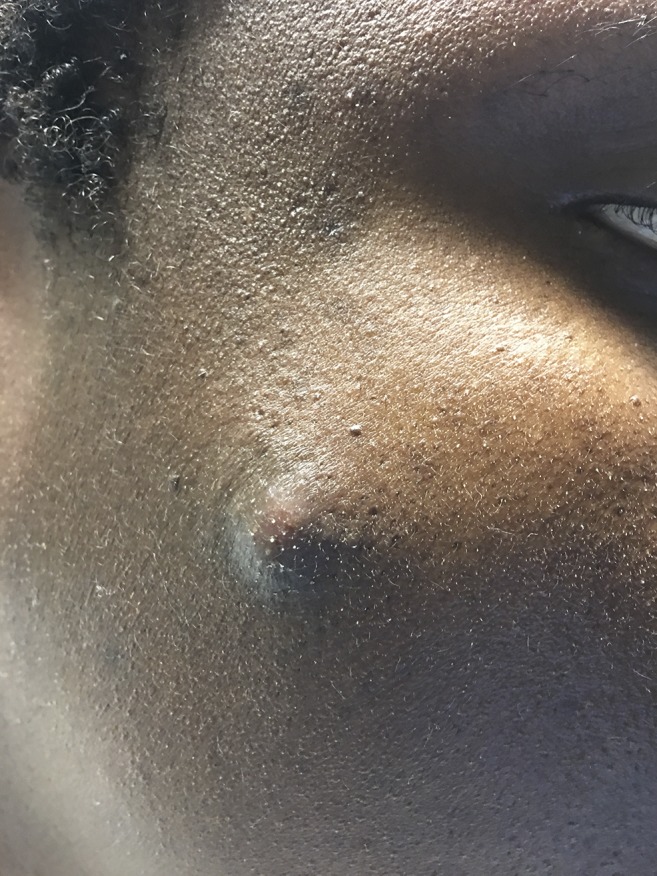
Right cheek cutaneous nodule, 1 × 1 cm. This figure appears in color at www.ajtmh.org.

**Figure 2.**
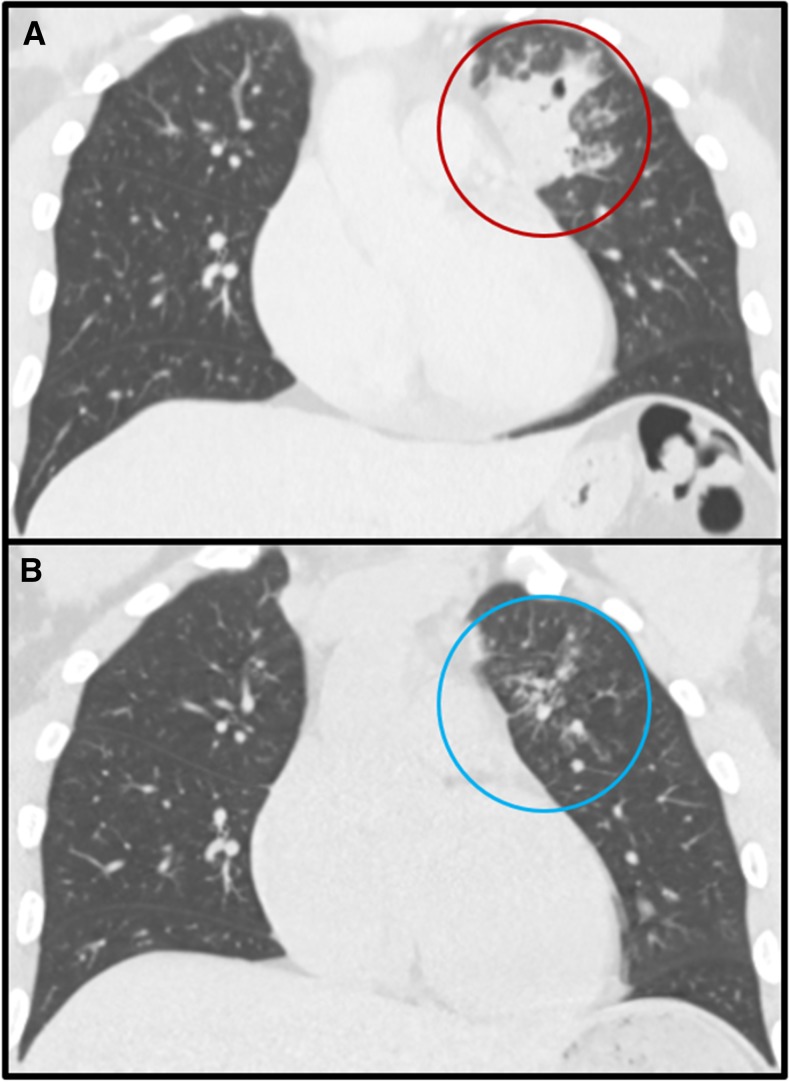
Computed tomography with intravenous contrast of the chest revealing left upper lobe opacity and cavitation due to coccidioidomycosis (**A**, red circle) with near resolution of lesion noted on repeat imaging after 6 months of fluconazole therapy (**B**, blue circle). This figure appears in color at www.ajtmh.org.

**Figure 3.**
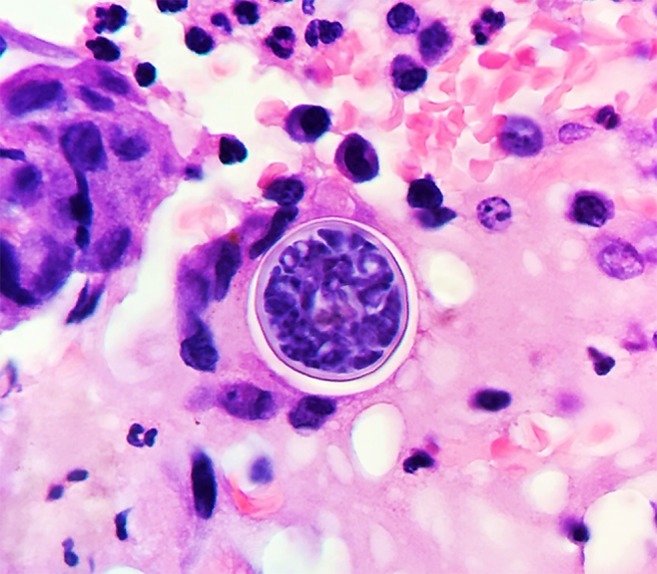
Coccidioidal spherule (30 µm), thick walled, with active segmentation and endosporulation, surrounded by granulomatous tissue seen on light microscopy (H&E staining) from tissue biopsy of the cutaneous nodule. This figure appears in color at www.ajtmh.org.

A 34-year-old man from Guinea, West Africa, presented to the hospital with a 2-week history of subjective fever, drenching night sweats, worsening dry cough, and profound fatigue. A non-tender skin nodule on his right cheek appeared 2 days earlier (Figure 1). He immigrated to Tucson, AZ, 8 years ago to study engineering. The patient denied any known history of tuberculosis, HIV infection, sick contacts, or significant outdoor exposure. He recently started working for a distribution center where he loaded and unloaded cargo from semitrailer trucks carrying freight. He had no family history of disease or immune deficiencies. Computed tomography (CT) of the thorax with intravenous contrast revealed a large consolidated opacity in the left upper lobe with evidence of early cavitation (Figure 2A). Laboratory findings were significant for leukocytosis (WBC 13.2 × 10^3^), eosinophilia (2.3 × 10^3^), and a negative HIV test. Before a punch biopsy, a small amount of purulent fluid was expressed from the skin nodule and sent for cultures. Three sputum analyses for acid-fast bacilli were negative and subsequent cultures yielded no mycobacterial growth. Serum 1,3 β-D glucan assay was positive at 460 pg/mL. Urine histoplasma antigen and blastomyces serologic testing were negative. Enzyme immunoassay tests for anti-coccidioidal immunoglobulin M and immunoglobulin G were positive. The anti-coccidioidal antibody complement-fixing (CF) titer was 1:4. Disseminated coccidioidomycosis was confirmed when the fungal culture from the skin nodule grew *Coccidioides* species and subsequent tissue histopathology showed spherules (Figure 3).

Coccidioidomycosis is caused by the endemic fungi *Coccidioides immitis* and *Coccidioides posadasii*. These are found in the soil of central and southern California, the low deserts of Arizona, and other regions throughout the western United States, Mexico, and Central and South America.^1^ Cutaneous granulomatous lesions represent the most common form of extrapulmonary coccidioidal dissemination. The Infectious Diseases Society of America 2016 coccidioidomycosis guidelines suggest that cutaneous dissemination warrants therapy.^1^ Oral fluconazole between 400 mg and 800 mg daily is the most common treatment; oral itraconazole 200 mg twice daily is an accepted alternative for treatment of cutaneous dissemination.^1^ It is unclear why certain populations are at higher risk than others for disseminated coccidioidomycosis. Studies have shown that risk factors for disseminated disease include black and Asian race/ethnicity, pregnancy, diabetes, HIV infection, active chemotherapy, and exogenous immunosuppression (e.g., prednisone, anti-graft rejection medications, and anti-inflammatory biologicals).^2–4^ A recent investigation has also revealed that functional and genetic defects in the interleukin-12/interferon-γ axis and signal transducer and activator of transcription 3 (STAT3)-mediated immunity may lead to a predisposition for disseminated coccidioidomycosis.^4^

The patient was placed on oral fluconazole 800 mg daily and discharged from the hospital. At his 2-week clinic follow-up visit, he reported decreased fever and night sweats, with continued improvement in his cough. After 6 months of oral fluconazole therapy, a repeat CT of the thorax with intravenous contrast revealed near resolution of the previous left upper lobe consolidation without any evidence of cavitation (Figure 2B). The patient was no longer complaining of any systemic symptoms. His previous skin nodule was no longer visible or palpable on the right cheek. A repeat anti-coccidioidal antibody CF titer was not checked because the patient had demonstrated clear clinical and radiologic improvement. The anti-coccidioidal antibody CF concentration is expected to decrease as the coccidioidal infection resolves, and rising CF titers can indicate there may be unresolved disease or ongoing dissemination.^1^ He will be continued on fluconazole for at least 1 year of therapy. Even with prolonged treatment, relapse is common after discontinuation of therapy.^1^ Clinicians should be aware that individuals of African descent who travel or live in areas endemic for *Coccidioides* species are at increased risk for disseminated disease.
